# Age‐stratified and gender‐specific reference intervals of six tumor markers panel of lung cancer: A geographic‐based multicenter study in China

**DOI:** 10.1002/jcla.23816

**Published:** 2021-05-12

**Authors:** Yan Li, Ming Li, Yi Zhang, Jianping Zhou, Li Jiang, Chen Yang, Gang Li, Wei Qu, Xinhui Li, Yong Chen, Qing Chen, Wei Wang, Shukui Wang, Jin liang Xing, Huayi Huang

**Affiliations:** ^1^ Department of Laboratory Medicine Renmin Hospital of Wuhan University Wuhan China; ^2^ Department of Laboratory Medicine The First Affiliated Hospital of University of Science and Technology of China Hefei China; ^3^ Department of Laboratory Medicine Qilu Hospital of Shandong University Jinan China; ^4^ Department of Radio Immunoassay Center Shaanxi Provincial People’s Hospital Xi’an China; ^5^ Department of Laboratory Medicine Sichuan Academy of Medical Sciences & Sichuan Provincial People's Hospital Chengdou China; ^6^ Department of Laboratory Medicine Suzhou Municipal Hospital Suzhou China; ^7^ Department of Laboratory Medicine Henan Provincial People's Hospital Zhengzhou China; ^8^ Department of Nuclear Medicine Nanjing First Hospital Nanjing Medical University Nanjing China; ^9^ Department of Nuclear Medicine Xiangya Hospital Central South University Changsha China; ^10^ Division of in vitro Diagnostics Shenzhen Mindray Bio‐Medical Electronics Corporation Shenzhen China; ^11^ State Key Laboratory of Cancer Biology and Department of Physiology and Pathophysiology Fourth Military Medical University Xi’an China; ^12^ Department of Surgical Oncology Roswell Park Comprehensive Cancer Center Buffalo NY USA

**Keywords:** carcinoembryonic antigen, human epididymis protein 4, lung neoplasms, progastrin‐releasing peptide, tumor biomarkers

## Abstract

**Background:**

Serum biomarkers have been widely adopted in clinical practice for assisting lung cancer diagnoses, therapeutic monitoring, and prognostication. The function of a well‐performing tumor biomarker depends on a reliable reference interval (RI) with consideration of the study subjects’ age, gender, and geographical location. This study aimed to establish a RI for each of 6 lung cancer biomarkers for use in the whole country of China on Mindray platform.

**Methods:**

The levels of serum 6 lung cancer biomarkers—namely progastrin‐releasing peptide (ProGRP), neuron‐specific enolase (NSE), squamous cell carcinoma antigen (SCC), carcinoembryonic antigen (CEA), cytokeratin‐19 fragment (CYFRA21‐1), and human epididymis protein 4 (HE4)—were measured utilizing the chemiluminescence immunoassay on the Mindray CL‐6000i platform following the laboratory standard operating procedures in apparently healthy Chinese individuals on large cohort, multicenter, and geographical consideration bases. The CLSI EP28‐A3C guideline was followed for the enrollment of study subjects.

**Results:**

The age‐stratified, gender‐specific RIs for ProGRP, NSE, SCC, CEA, CYFRA21‐1, and HE4 lung cancer biomarkers in the Chinese population have been established as described in the results and discussion in this work. In addition, various levels of the six lung cancer biomarkers among nine geographical locations in China have been observed.

**Conclusions:**

The sample volume of study cohort, age, and geographical location should be considered upon establishing a reliable biomarker RI. A RI for each of six lung cancer biomarkers has been established. The results from this study would be helpful for clinical laboratories in interpreting the analytical results and for clinicians in patient management.

## Graphical abstract

A total of 2259 apparently healthy subjects (ages 13–87) from 9 tier‐3 hospitals in China (Zhengzhou, North; Xi'an, Northwest; Jinan, Hefei, Nanjing, and Suzhou, East; Wuhan, Central; Changsha, Central South; Chengdu, Southwest); CLSI C28‐A3 guidelines was followed; Chemiluminescent immunoassay (Mindray CL‐2000i and CL‐6000i) for ProGRP, NSE, SCC, CEA, CYFRA21‐1, and HE4; SPSS statistical software was used to analyze the data; Age‐stratified, gender‐specific, and geographic‐based reference intervals have been established.

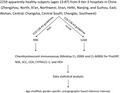

## 1 INTRODUCTION

Lung cancer is the most common neoplasm both in incidence and mortality worldwide, including in China.^1^, ^2^, ^3^, ^4^ Globally, the lack of reliable tools for early screening, diagnosis, and treatment monitoring has resulted in late‐stage or terminal diagnoses. Low dose computed tomography (LDCT) and tumor markers are common tools currently available for lung cancer diagnosis in clinical practice. The procedure, however, has a high false positive rate, limiting its efficacy in helping to identify cancer. To date, several tumor markers have been used for lung cancer screening, diagnoses, therapeutic monitoring, and prognostication in clinics, with the assay procedure being minimally invasive, convenient, and easy to access with low costs in clinical practice.^5^, ^6^ Elevation of CEA has been found in many types of diseases, including lung cancer, with lung cancer being more specific for adenocarcinoma of the lung.^7^, ^8^ Elevation of CYFRA21‐1 has been found to be associated with worse five‐year overall survival and local regional relapse‐free survival in non‐small cell lung cancer (NSCLC).^9^, ^10^ NSE is considered a marker for small cell lung cancer invasiveness.^11^ Squamous cell carcinoma antigen (SCC) has been considered as a squamous cell carcinoma specific marker.^12^ HE4 is usually considered as a biomarker for ovarian cancer and used in the diagnosis of a neoplasm. Elevation of HE4 in serum and pleural effusion were found in NSCLS patients, making it a potential new lung cancer biomarker.^13^, ^14^

To date, the sensitivity and specificity of lung cancer biomarkers are still a bottleneck to overcome in lung cancer screening and diagnosis. There is thus an urgent need to improve lung cancer risk assessments because current in vitro diagnosis‐based screening criteria miss a large number of cases.^15^ Although there are plenty of reports regarding lung cancer biomarkers and their diagnostic performance available thus far,^16^, ^17^, ^18^, ^19^, ^20^, ^21^, ^22^, ^23^ a well‐established reference interval for each lung cancer biomarker is still desired to enhance the performance of the aforementioned biomarkers. To establish a well‐designed and dependable reference interval, certain criteria of the study subjects—such as age, gender, geographic location, and life style—should be considered, because they may have an impact on the levels of biomarkers of individuals to be investigated. The sample volume to be enrolled in the study is another important factor when establishing a well‐performed reference interval. Although CLSI^24^ requires a minimum of 120 samples to satisfy the sample volume in establishing a reference interval considering the cost of conduct, a larger volume sample will result in a better Poisson distribution and represent a “near‐true” value in the population, if the budget allows. To conduct a reference interval study for a tumor biomarker, a standardized evaluation of tumor markers on a large population with age‐stratified, gender‐specific, and geographic location well represented of healthy subjects are desired. Lastly, establishing a reference interval with a multi‐marker panel of lung cancer biomarkers in multiple hospitals simultaneously is a challenge. We report the establishment of a reference interval for six individual lung cancer biomarkers, namely the progastrin‐releasing peptide (ProGRP), neuron‐specific enolase (NSE), squamous carcinoma antigen (SCC), carcinoembryonic antigen (CEA), cytokeratin‐19 fragment (CYFRA21‐1), and human epididymis protein 4 (HE4) as phase I of our recent multi‐center clinical study series with age‐stratified, gender‐specific, large cohort, and geographic population considerations from 9 large tier‐3 hospitals in China.

## 2 METHODS

### 2.1 Study design and ethic approval

The design of this study was based on the CLSI EP28‐A3C “Defining, Establishing, and Verifying Reference Intervals in the Clinical Laboratory; Approved Guideline‐Third Edition”.^24^ Laboratory parameters from individuals who visited the health examination center for routine health checks in all participating hospitals were collected. These subjects were provided with a health condition questionnaire before blood collection in order to meet the requirement from CLSI EP28‐A3C. The exclusion criteria including smoking, alcoholism, medication, diabetes, any cancer or cancer history, any known infection, hypertension, abnormal kidney function, anxiety, recent hospitalization, family inherited diseases, menstruation period, lactation, pregnancy, and use of vitamin supplements. The enrolled study subjects’ name, gender, age, and medical record number were also collected.

The study was carried out under the permission and approval from the Institutional Review Board (IRB) / Ethics Committee of all participating hospitals.

### 2.2 Study site selection

Nine large tier‐3 hospitals were selected, representing North, Northwest, Southwest, Central, Central South, and East China.

### 2.3 Sample collection and storage

Fasting blood was collected from all ostensibly healthy individuals visiting the health examination center of a participating hospital who met the requirements of the study questionnaire. A serum collecting tube, routinely used in each participating hospital, was used to collect the blood, and the samples were transferred to the clinical laboratory for processing by a qualified technician to isolate the serum. The collected serum was then stored at −80 ^O^C for a period of 1–3 months, until required.

### 2.4 Chemiluminescent immunoassay of tumor biomarkers

ProGRP, NSE, SCC, CEA, CYFRA21‐1, and HE4 were analyzed on a Mindray CL‐2000i or CL‐6000i Chemiluminescent immunoassay analyzer (Mindray Bio‐Medical Electronics Corporation, Shenzhen, Guangdong, China) following the manufacturer's instructions. Results were deposited in the Laboratory Information System to be further analyzed.

### 2.5 Statistical analyses

#### 2.5.1 Determination of outliers by Dixon's test

According to the CLSI C28‐A3 guidelines and the principle of statistics,^24^, ^25^ the outliers were identified and removed following a report from Liu et al.^25^ Specifically, Dixon's test was used to remove the outliers in the datasets following CLSI C28‐A3 and Liu et al..^24^, ^25^ The outliers were determined by a D/R ratio in Dixon's test, where D is the absolute difference between an extreme observation (large or small) and the next largest (or smallest) observation, and R is the range of all observations, including extremes. If D/R ≥ 1/3, then the specific data will be removed.

#### 2.5.2 Normality test of datasets

The distribution of datasets of 6 individual lung cancer biomarkers of 9 participating hospitals was analyzed using One‐Sample Kolmogorov‐Smirnov Test, a *p* value <0.05 is considered significant in difference. This analytical result will determine whether parametric or non‐parametric statistical method will be used in next step analysis by SPSS version 18.0 software.

#### 2.5.3 Transformation of skewed data

After normality test, the skewed distribution (non‐normal distribution) was transformed into normal distribution by using the Box‐Cox method.

#### 2.5.4 Sub‐classification determination for reference interval establishment

Two common factors are considered when establishing a RI, the sub‐classification (subgrouping) based on gender and age. In this work, the recommendation from CLSI C28‐A3 of Establishment of Reference Interval for Clinical Laboratory Test Items was followed, and the *Z* test was used to determine whether sub‐classification is needed for each tumor biomarker. By definition, if *Z* > Z*, then the difference between the RIs is statistically significant (*p* < 0.05) between two groups, thus a RI for each group is needed. In other words, if *Z* < Z*, then the difference between the two RIs is not statistically significant (*p* > 0.05), and the RIs can be combined.^24^, ^25^ However, when the *Z* value >Z* between gender of a specific biomarker, the sub‐classification of age should be also performed regardless of the *Z* value. In this study, we have grouped the age into > = 50 and <50 groups only considering the sample size to be met the minimum of 120 based upon the CLSI guidelines as well as the fact that most of lung cancer occurred in the elderly people.

#### 2.5.5 Production of reference intervals for 6 lung cancer biomarkers

To establish a RI for each of six lung cancer biomarkers, following CLSI C28‐A3 guidelines and data process are described above. A 95% percentile was presented for the upper scale of the RI, and a 90% confidence interval (CI) was also displayed.

## 3 RESULTS

The basic information of healthy subjects is listed in Table 1.

**TABLE 1:** Basic Information of Healthy Subjects

Classification	n	Median (range)
Total	2259	
Male	990	52 (14–85)
Female	1269	52 (13–87)
Age		
18–29 years	104	52 (13–87)
30–49 years	828	
≥50 years	1330	

### 3.1 Normality test results of datasets of 6 individual lung cancer biomarkers from 9 participating hospitals

The distributional pattern of 6 individual lung cancer biomarkers of 9 participating hospitals and pooled datasets of all 9 hospitals was analyzed as displayed in Supplementary Figures S1–S7, in which Figures S1–S6 represent datasets of individual hospital, while Figure S7 represents pooled dataset of all 9 hospitals for each biomarker. Table 2 shows the normality test (One‐Sample Kolmogorov‐Smirnov Test) results of 6 individual biomarkers. Results indicate that datasets from all biomarkers are skewed distribution (*p* < 0.05 for all). Thus, all the data have been transformed into normal distribution by using the Box‐Cox method.

**TABLE 2:** Normality Test Results (One‐Sample Kolmogorov‐Smirnov Test)

	ProGRP	NSE	SCC	CEA	CYFRA21‐1	HE4
n	2259	2256	2258	2256	2259	2258
Normal Parameters						
Mean	39.48	13.69	0.87	1.73	1.99	47.18
STDEV	15.19	4.93	0.44	1.20	0.96	19.49
Most Extreme Differences						
Absolute	0.055	0.107	0.108	0.118	0.105	0.137
Positive	0.055	0.107	0.108	0.118	0.105	0.137
Negative	−0.039	−0.056	−0.087	−0.113	−0.091	−0.110
Kolmogorov‐Smirnov *Z*	2.593	5.080	5.142	5.582	5.010	6.523
Asymp. Sig. (2‐tailed)	0.000	0.000	0.000	0.000	0.000	0.000

### 3.2 Sub‐classification determination for reference interval establishment based upon gender and age (*Z* test)

Tables 3 and 4 show the statistical results for determination of sub‐classifying for RI establishment based on gender and age following the CLSI C28‐A3 guidelines using a *Z* test. Results indicate that ProGRP and CYFRA21‐1 require 2 RIs to represent each age group (age <50 years and > = 50 years) because the *Z* value is greater than Z* value. For NSE, there is no need to perform sub‐classification since the *Z* value is smaller than Z*. Since SCC had a *Z* > Z* in sub‐classification, thus it requires 2 RIs to represent each gender group and 2 RIs for age sub‐grouping regardless of *Z* and Z* values; CEA and HE4 require 4 RIs to represent gender and age groups, respectively.

**TABLE 3:** Sub‐classification Determination for Reference Interval Establishment Based On Gender (*Z* test)

	Gender	n	Mean	STDEV	*Z*	*Z*
ProGRP	M	990	40.05	14.07	2.343	9.204
	F	1267	38.84	15.25		
NSE	M	989	13.93	4.24	4.501	9.204
	F	1267	13.51	5.41		
SCC	M	989	0.98	0.46	13.553	9.202
	F	1269	0.77	0.40		
CEA	M	989	2.05	1.31	13.238	9.198
	F	1267	1.48	1.05		
CYFRA21‐1	M	990	2.10	0.90	7.077	9.204
	F	1269	1.90	1.00		
HE4	M	986	52.42	19.95	14.273	9.202
	F	1269	42.57	14.09		

Abbreviation: STDEV, standard deviation.

**TABLE 4:** Sub‐classification Determination for Reference Interval Establishment Based Upon Age (*Z* test)

	Gender	Age	n	Mean	STDEV	*Z*	*Z*
ProGRP	M+F	<50	932	35.84	11.76	−15.922	6.093
		> = 50	1325	43.10	14.81		
NSE	M+F	<50	932	14.31	4.56	−5.216	6.093
		> = 50	1324	13.66	3.97		
SCC	M	<50	415	0.96	0.43	−1.082	6.090
		> = 50	574	1.00	0.48		
	F	<50	517	0.78	0.41	0.394	6.898
		> = 50	752	0.77	0.39		
CEA	M	<50	415	1.73	1.11	−7.520	6.090
		> = 50	574	2.27	1.39		
	F	<50	516	1.11	0.69	−12.163	6.893
		> = 50	751	1.73	1.18		
CYFRA21‐1	M+F	<50	932	1.93	0.73	−9.882	6.093
		> = 50	1327	2.22	0.98		
HE4	M	<50	414	43.84	13.35	−14.387	6.090
		> =50	572	58.64	21.57		
	F	<50	517	37.92	9.99	−11.648	6.898
		> = 50	752	45.76	15.54		

### 3.3 Determination of reference intervals and 90% confidence intervals of 6 lung cancer biomarkers with age‐stratified, gender specific, and geographical consideration

Table 5 shows the RIs generated for 6 biomarkers based on CLSI C28‐A3 recommendation.

**TABLE 5:** Reference Intervals for 6 Lung Cancer Biomarkers (Non‐Parametric Rank Method, with P95 and 90% CI on the Basis of CLSI C28‐A3 Guidelines)

	Gender	Age	n	Median	RIs	90%CI
ProGRP	M+F	<50	932	32.53	0–54.81	52.55–57.58
		> = 50	1325	41.93	0–70.69	68.05–72.74
NSE	M+F		2256	13.00	0–22.66	21.79–23.36
SCC	M	<50	415	0.96	0–1.66	1.52–1.78
		> = 50	575	1.05	0–1.92	1.80–2.11
	F	<50	517	0.78	0–1.35	1.28–1.53
		> = 50	752	0.77	0–1.46	1.38–1.52
CEA	M	<50	415	1.53	0–3.57	3.29–3.95
		> = 50	574	1.98	0–4.93	4.50–5.13
	F	<50	516	0.93	0–2.46	2.21–2.69
		> = 50	751	1.44	0–3.61	3.45–3.92
CYFRA21‐1	M+F	<50	932	1.62	0–3.10	2.98–3.19
		> = 50	1327	1.90	0–3.90	3.63–4.06
HE4	M	<50	414	40.89	0–69.63	64.04–75.60
		> = 50	572	53.94	0–97.66	92.66–110.18
	F	<50	517	37.04	0–55.84	53.24–57.09
		> = 50	752	43.71	0–70.04	67.23–74.62

Note

M+F, mixed gender with no need a separate RI.

Abbreviations: CI, confidence interval; RIs, reference intervals.

**FIGURE 1:**
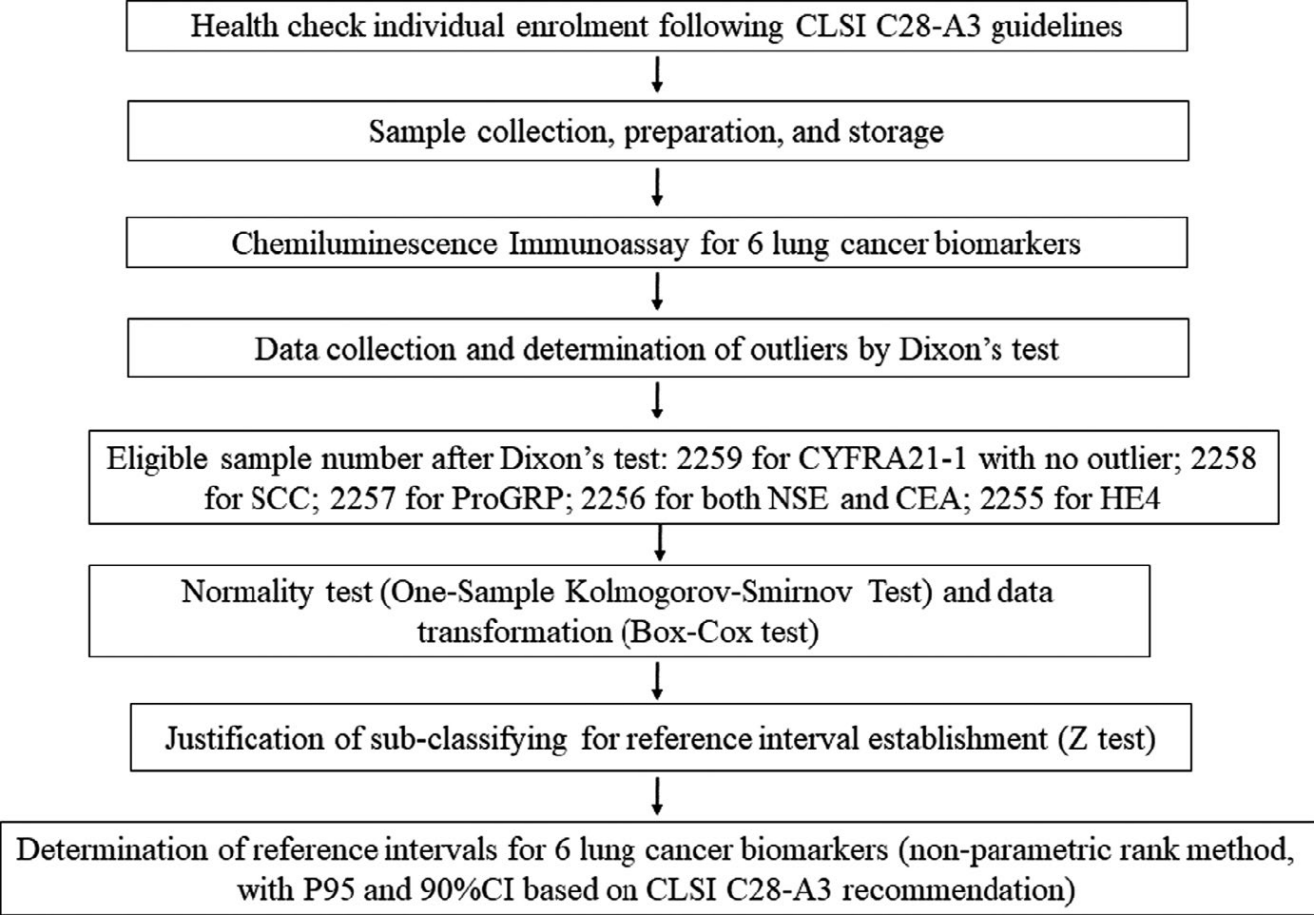
Flow chart of establishing a reference interval for ProGRP, NSE, SCC, CEA, CYFRA21‐1, and HE4 lung cancer biomarkers in Chinese population

All the above data analysis flow chart is displayed in Figure 1.

## 4 DISCUSSION

Since the performance of lung cancer biomarkers is still debatable in clinical practice, their use for lung cancer diagnosis, therapeutic monitoring, and prognosis prediction is ambiguous, of which is partly because of lacking a rigorous standardized reference interval.^17^, ^18^, ^19^, ^20^, ^21^, ^22^, ^23^, ^24^, ^25^, ^26^, ^27^, ^28^, ^29^, ^30^, ^31^, ^32^, ^33^ A reliable reference interval is therefore critical for the performance evaluation of a biomarker. Apart from following the requirements of CLSI EP28‐A3C guidelines, this study also considered geography to explore whether physical location influenced outcomes when establishing a reference interval for biomarkers. By doing so, the following information was compiled, enabling us to discuss the merits of a specific reference interval, which is supposed to be considered in clinical laboratory.

### 4.1 Study subjects enrolled in this study

As indicated in Table 1, a total of 2259 ostensibly healthy individuals were enrolled in this study, meeting the requirements of CLSI. In fact, each participating hospital in this study enrolled more than 120 samples. Sample size is a critical factor when establishing a RI. Although the CLSI requires a minimum of 120 samples, however, the larger the sample size is, the better distribution it will obtain in statistical analysis which means better in representation. Thus, our study enrolled more than 120 samples in each hospital.

### 4.2 Normality testing of datasets and data transformation

The purpose of normality test is to evaluate if the sample distribution is normal or not. If the sample distribution is normal, then the parametric method of statistical analysis will be used. In other words, if sample distribution is not normal (skewed distribution), then the non‐parametric method of statistical analysis should be used to obtain adequate results. In this study, the results revealed that the datasets of 6 biomarkers were skewed distribution. Thus, the data was transformed into normal distribution by using the Box‐Cox method for the following *Z* test.

### 4.3 Application of *Z* test and the results interpretation

As mentioned above, the *Z* test is applied only when the data is normally distributed according to the CLSI guidelines. Thus, the datasets which showed skewed distribution were transformed into normally distributed data by using the Box‐Cox method prior to the *Z* test. *Z* test defines whether or not separate RIs are required for each of 6 biomarkers with gender and age sub‐classification. ProGRP and CYFRA21‐1 require 2 RIs to represent two age groups; CEA and HE4 require 4 RIs to represent both gender and age groups. For SCC, although the *Z* test results indicated that there was only a *Z* > Z* between genders, however, the RIs for two age groups are still required regardless of the *Z* values between age groups, thus the total RIs required for SCC are 4. There is no need to perform sub‐classification for NSE which means 1 RI is applicable for all ages and both genders. This work is to minimize the unnecessary cost and for the ease of application in clinical practice.

### 4.4 Production of reference intervals of lung cancer biomarkers ProGRP, NSE, SCC, CEA, CYFRA21‐1, and HE4 with age‐stratified, gender specific, and geographic consideration

After calculating, RIs for each of 6 biomarkers with 95 percentiles and 90% CI have been established. Specifically, for ProGRP, 2 separate RIs for age <50 (0–54.81) and age >=50 (0–70.69) will be used with no need gender sub‐classification. For NSE, there is no need to perform gender and age sub‐classification according to *Z* test results. Thus, only 1 RI is needed for NSE (0–22.66). For SCC, 4 RIs represent for gender and age sub‐classification are as following: the RIs for male <50 years and >= 50 years are 0–1.66 and 0–1.92, respectively, while the RIs for female <50 years and >= 50 years are 0–1.35 and 0–1.46, respectively. CEA requires 4 RIs for both gender and age sub‐groups. The RIs for male <50 years and >= 50 years are 0–3.57 and 0–4.93, respectively; while the RIs for female <50 years and >= 50 years are 0–2.46 and 0–3.61, respectively. Similar to ProGRP, 2 RIs are required for CYFRA21‐1 with age sub‐classification, the RI for both male and female with age <50 is 0–3.10, while the RI for age >=50 is 0–3.90. Lastly, similar to CEA, HE4 requires 4 RIs for both gender and age sub‐groups. The RIs for male <50 years and >= 50 years are 0–69.63 and 0–97.66, respectively, while the RIs for female <50 years and >= 50 years are 0–55.84 and 0–70.04, respectively. These RIs represent associated biomarkers intended for use on Mindray's platform in China. It is noteworthy that studies regarding RIs for lung cancer biomarkers were reported previously. ^25^, ^26^, ^27^, ^28^, ^29^, ^30^, ^31^ For instance, Liu et al described a RI for NSE performed on a Roche Cobas e602 platform based on a large sample size of Chinese population. However, the study was a single laboratory observation and for only one biomarker.^25^ While Yang et al reported RIs for CEA, NSE, and CYFRA21‐1 from a multi‐centric study in Henan Province in Northern China using a Roche e601 platform, also with large sample size. However, the study did not establish the RIs for SCC, ProGRP, and HE4 as yet, and the results represented for use in laboratories in Henan Province.^26^ Yang et al reported a RI for ProGRP based on a relatively small sample size for a single laboratory in South‐central China, also performed on a Roche e601. ^27^ Similarly, Zhu et al reported a RI for ProGRP from a rather large sample size with different age groups performed on a Roche e601 platform for a single laboratory in Southwest China.^28^ Our study represents multi‐centric and large sample size with geographic location consideration of the country of China. This study also considered age and gender stratification. Furthermore, the RIs are intended to use on the Mindray chemiluminescence immunoassay platform in China. Lastly, our study established RIs for 6 lung cancer biomarkers simultaneously.

## 5 CONCLUSIONS

An age‐stratified, gender‐specific, and geographical considered reference interval has been established in Chinese population for 6 individual lung tumor biomarkers which can be used on Mindray chemiluminescence immunoassay platform in clinical laboratory practice in China.

### 5.1 Limitations of this study

It is worthy to point out some limitations of this study: (1) sample size could be larger if the work‐flow was performed more efficiently and rigorously in subject enrollment during the study; (2) age and gender matching in subjects enrollment in individual participating hospital and among hospitals could be controlled better, thus avoiding the bias in statistical results; (3) multi‐platform comparison is an ideal work in the future effort which is lacking in this study due to budget issue; (4) following‐up of those individuals who had elevated serum biomarker (s) is an interesting task to conduct which is lacking in this study also.

## CONFLICT OF INTEREST

Mindray Corporation provided the chemiluminescence immunoassay analyzer and reagents to the participating hospitals. A proportion of intellectual property right will be shared by Mindray Corporation and the participating hospitals.

## AUTHOR CONTRIBUTIONS

Li Y, Li M, Zhang Y, Zhou J, Jiang L, Yang C, Li G, Qu W, and Li X: involved in organizing and conducting laboratory analysis, data collection, and discussion of the manuscript. Chen Y, Chen Q, and Wang W: involved in data collection and statistical management. Wang S and Xing J‐L: involved in project conception and discussion. Huang H: involved in project conception and manuscript writing.

## Supporting information

Fig S1‐S7

## Data Availability

The datasets generated during and/or analyzed during the current study are available from the corresponding author on reasonable request.
