# Supplementary material for: Age‐stratified and gender‐specific reference intervals of six tumor markers panel of lung cancer: A geographic‐based multicenter study in China
Source: J Clin Lab Anal. 2021 May 12;35(6):e23816. doi: 10.1002/jcla.23816 (PMC8183943; doi:10.1002/jcla.23816)
Supplement: Supplementary file 1 — Fig S1‐S7 [file JCLA-35-e23816-s001.docx]

**FIGURE S1 Distribution histogram of 6 lung cancer biomarkers from 9 participating hospitals (ProGRP)**

**FIGURE S2 Distribution histogram of 6 lung cancer biomarkers from 9 participating hospitals (NSE)**

**FIGURE S3 Distribution histogram of 6 lung cancer biomarkers from 9 participating hospitals (SCC)**

**FIGURE S4 Distribution histogram of 6 lung cancer biomarkers from 9 participating hospitals (CEA)**

**FIGURE S5 Distribution histogram of 6 lung cancer biomarkers from 9 participating hospitals (CYFRA21-1)**

**FIGURE S6 Distribution histogram of 6 lung cancer biomarkers from 9 participating hospitals (HE4)**

**FIGURE S7 Distribution histogram of 6 lung cancer biomarkers from 9 participating hospitals (pooled)**
